# Delivery and evaluation of simulations to promote authentic and meaningful engagement in childhood disability research

**DOI:** 10.1186/s40900-023-00468-9

**Published:** 2023-07-18

**Authors:** Samantha K. Micsinszki, Nadia L. Tanel, Julia Kowal, Gillian King, Dolly Menna-Dack, Angel Chu, Kathryn Parker, Michelle Phoenix

**Affiliations:** 1 School of Rehabilitation Science, McMaster University, Hamilton, ON Canada; 2 CanChild Centre for Childhood Disability Research, McMaster University, Hamilton, ON Canada; 3 Holland Bloorview Kids Rehabilitation Hospital, Toronto, ON Canada; 4 Department of Occupational Science and Occupational Therapy, University of Toronto, Toronto, ON Canada; 5 Centre for Advancing Collaborative Healthcare and Education (CACHE), University of Toronto, Toronto, Canada; 6 Department of Paediatrics, Temerty Faculty of Medicine, University of Toronto, Toronto, Canada

**Keywords:** Simulation, Evaluation, Patient engagement, Family engagement, Patient-oriented research, Childhood disability, Authentic, Meaningful

## Abstract

**Background:**

In 2019, our interdisciplinary team of researchers, family members, and youth co-designed four simulation training videos and accompanying facilitation resources to prepare youth, family members, trainees, and researchers to build the knowledge and skills to engage in patient-oriented research (POR) authentically and meaningfully. Videos covered challenges in aspects of the research process including (1) forming a project team; (2) identifying project objectives and priorities; (3) agreeing on results; and (4) carrying out knowledge translation.

**Methods:**

The purpose of the study was to deliver four simulation training videos across 2 two-hour facilitated workshops with researchers, trainees, and family partners. We evaluated whether the training videos and facilitated discussion of the simulations helped to improve knowledge and attitudes about authentic and meaningful partnership in research and self-perceived ability to engage in POR. An explanatory sequential two-phase mixed methods design was used. Phase 1 (quantitative) included two training workshops and a pre/post-training survey. Phase 2 (qualitative) included two qualitative focus groups. Results of each phase were analyzed separately and then combined during interpretation.

**Results:**

Sixteen individuals (including researchers/research staff, trainees, family members, clinicians) took part in this research study. Overall, participants were highly receptive to the training, providing high scores on measures of acceptability, appropriateness, and feasibility. While the training videos and facilitated discussion of the simulations were found to increase participants’ knowledge and ability to engage in authentic and meaningful POR, we found no significant change in attitude or intent. Recommendations about the simulation content and delivery were provided to inform for future use.

**Conclusions:**

The simulations were found to be a positive and impactful way for collaborative research teams to build knowledge and ability to engage in authentic and meaningful POR. Recommendations for future work include covering different content areas with varying levels of nuance; and offering the training to stakeholders in a variety of roles, such as those higher-ranked academic positions.

**Supplementary Information:**

The online version contains supplementary material available at 10.1186/s40900-023-00468-9.

## Abstract (plain language summary)

In 2019, our team of researchers, family members, and youth worked together to design and develop four digitally recorded simulation videos that can be used to train youth, caregivers/families, trainees, and researchers to engage with each other in research so that all parties feel supported and valued. This paper describes how the four simulation videos were packaged in the training and then delivered to 16 participants (researchers, trainees, and caregivers/families). We used multiple ways to evaluate the videos and training, including a survey before and after the training, focus groups with participants after the training, and written reflections shared by the training facilitators after the training was finished. We found that the simulation videos increased participants’ knowledge on engagement and their self-reported ability to engage in authentic and meaningful patient-oriented research. Participants rated their belief in engagement and their intent to engage in collaborative research highly at the pre-test and this remained consistent at the post-test. Participants liked that the simulations focused on challenges in research engagement and that the training was offered to researchers and family partners together. They provided valuable feedback on what we should change about the simulations, including the content, which should have less exaggerated lessons and to add more topics. They also suggested it would be helpful if stakeholders other than just the research team complete the training in the future, especially those who are in higher positions of academic power.

**Supplementary Information:**

The online version contains supplementary material available at 10.1186/s40900-023-00468-9.

## Background

### Meaningful engagement in patient-oriented research

Engagement is a central component of patient-oriented research (POR) and refers to “meaningful and active collaboration in governance, priority setting, conducting research and knowledge translation” [1, (p5)] where patients and their families can be involved in all aspects of the research process, from idea conceptualization and research design through to dissemination [2]. Including patients who have personal (lived) experience of a health condition as patient partners (and their families) in various roles (e.g., consultants, collaborators, co-investigators etc.) on research teams is increasingly requested by health research organizations and their funders and is intended to advance research that is relevant and focused on the priorities of the communities being represented [3–6]. When engagement is meaningful (i.e., “planned, supported and valued involvement of patients in the research process” [7, (p404)]), patients and their families are invited to engage as more than just a ‘tick box’ and are provided meaningful opportunities to contribute [8].

### Training opportunities in patient-oriented research

Developing patient engagement resources is an important step in supporting authentic and meaningful engagement in research [9]. While there are many identified benefits of POR for the research process (e.g., improved study enrollment and decreased attrition) [10, 11] and for patient partners (e.g., improved research skills, internal validation, and increased confidence and self-esteem) [12–14] recent research has identified that researchers and patient partners may lack the understanding and skills to engage meaningfully in research partnerships [2, 13]. When researchers and patient partners lack the skills needed to engage meaningfully in research partnerships, this can lead to tokenistic, inauthentic, and failed partnerships [13, 15–17]. A few strategies have been identified to optimize engagement and ensure successful partnership [10], including: (1) educating and training for both patient partners and researchers/ investigators; and (2) building an environment that supports trust, respect, reciprocity, and co-learning [2]. A 2016 report from the British Columbia Strategy for Patient-Oriented Research (SPOR) Support Unit indicated that there was a lack of training available to support authentic and meaningful partnerships [18]. Since that time, training programs have begun to surface across Canada and internationally. In Canada, we are aware of at least three, namely, Patient Oriented Research Curriculum in Child Health (PORCCH) [19]; Kids Brain Health Network, CanChild, McMaster Continuing Education Family Engagement in Research (FER) Course [20]; and Patient and Community Engagement Research (PACER) [21], all of which are innovative and comprehensive training programs designed to enhance and optimize success of involving patient partners in the research process.

### Co-learning

Patient partners and researchers have reported on the benefits of co-learning in research projects that include patients and caregivers as members of the research team [22]. These benefits include opportunities to develop empathy, trust, and mutual respect by learning about one another’s experiences and viewpoints, developing communication skills (e.g., decreased use of jargon or stigmatizing language, pre-circulation of meeting materials), and creation of “creativity, inclusivity, and cooperative ethos within our team, with plenty of space for kindness and laughter along the way” [22, (p4)]. These benefits of co-learning have also been observed in training available to community partners and researchers to prepare for participatory research project [23]. This training can build community capacity for partnership in research and the co-learning model explicitly aims to develop a culture of trust, open sharing of perspectives, and equality among all participants [23]. Despite these benefits of co-learning in POR training, most of the training programs available offer training to either the patient or researcher separately, except for the FER course which provides mixed group training with patients and researchers/trainees. It is recommended that co-learning opportunities about engagement in research include active participation, reflection, and evaluation of the experience [23].

### Simulation training

One active learning modality that has been widely used in medical education and evaluation is simulation training [24]. In simulation teaching and learning, a lifelike situation is presented that mirrors challenging professional events or experiences, offering the learner an opportunity to respond as they would in a natural environment without the risks that occur in a real situation, and to reflect and receive feedback on their performance [24, 25]. Simulations allow for diverse content areas to be targeted, the level of difficulty to be altered, for repeated practice, and for small group learning in a controlled environment that can be tailored to individual learning goals while providing opportunities for group members to learn from each other’s perspectives [24, 26]. While simulation has diverse applications, from flight simulation to medical procedures, we are most interested in simulations that include standardized patients to illustrate interpersonal challenges that arise in children’s rehabilitation clinical and research environments, such as communication, collaboration, problem solving, and addressing power imbalances [26, 27]. In children’s rehabilitation research there is likely to be a power difference between the patient, family members and the clinician or researcher [3], family-centred care and research require investigation of the client priorities and expectations for engagement [8], and sensitive topics are often addressed [28]. Therefore, the skills that can be targeted through simulation are particularly relevant for engagement in childhood disability research.

### Context for the current research: previous work completed by our team

In January 2019, our multidisciplinary team of researchers, trainees, research staff, clients, families, and educators held a one-day event to co-design the simulation videos. Details of the development of the simulations are reported by Micsinszki et al. [8] Briefly, part one of the simulation co-design involved one to two representatives from each of the five stakeholder groups (i.e., researchers and research staff, trainees, youth with disabilities, and parents/caregivers of a child with a disability) to share a story about a pre-circulated prompt related to an identified challenge in POR (e.g., identifying project objectives). As stories were specifically focused on challenges in POR, the simulation videos intentionally depict complex and difficult scenarios. In part two, Standardized Patient (SP) actors, individuals who are trained to portray various patient experiences [29] which may include patient and caregivers’ interactions with researchers and research partnerships, joined the simulation groups to help bring the scenarios to life and create the final scenario that would be depicted in the videos. Aspects of the scenarios depicted in the videos were slightly exaggerated intentionally (e.g., requesting a family partner one week prior to a grant deadline) to provoke stronger emotions and deeper discussion about the simulations.

Facilitator guides were developed by the research team to accompany each simulation video. These guides allow a facilitator to use the videos when training research teams that include representation from different stakeholder groups (i.e., childhood disability researchers and research staff, trainees, youth with disabilities, and parents/caregivers of a child with a disability). The simulation videos and guides are freely available online via the Holland Bloorview Kids Rehabilitation Hospital Simulation Hub [32]. These simulation videos are for educational, non-commercial purposes and, while facilitation experience is recommended to deliver the simulations, expertise in simulation methodology or delivery is not needed.

While previous work evaluated the codesign of the simulation videos and guides [8], it was necessary to also evaluate the application of these videos and guides in training for youth, families, and researchers. The purpose of the present study was therefore to deliver the four simulation videos and evaluate whether use of the simulations and facilitated discussion helped to build competency to engage meaningfully in POR among youth with disabilities, caregivers/families of children with disabilities, and researchers. The research objectives were to: (1) Describe knowledge, attitudes, and self-perceived ability regarding authentic and meaningful research engagement and determine if there was a change in each; (2) Determine how likely participants are to apply the knowledge gained from participating in the simulation training in their future research work; (3) Determine the extent to which the simulation training workshops and facilitation guides are an acceptable, appropriate, and feasible training tool; and (4) Provide recommendations for other research teams and/or organizations with teams that include individuals who represent a mix of participants who are interested in building capacity to authentically and meaningfully engage in POR.

## Methods

### Ethics approval and consent to participate

Institutional ethics approval (REB#0415) was received from Holland Bloorview Kids Rehabilitation Hospital. Written informed consent was obtained from all individuals who participated in this study.

### Study design

This research used an explanatory sequential two-phase mixed methods design [30]. This evaluation was reported according to the Guidance for Reporting Involvement of Patients and the Public (GRIPP) 2.0 [31] (see Additional file 1).

### Sample and recruitment

Participants included (1) PhD-trained childhood disability researchers affiliated with a university in Canada and/or academic teaching hospital; (2) masters, PhD, or postdoctoral trainees whose primary research focus was in the field of childhood disability and were supervised by a researcher at a university in Canada; (3) research staff (e.g., research assistants, research coordinators etc.); and (4) parents or primary caregivers of an individual up to the age of 29 with a childhood onset disability or acquired injury. Youth between the ages of 16 and 29 with a childhood disability or acquired injury were eligible to participate, however we were unable to recruit youth. Participants could hold multiple roles, such as researchers who are also clinicians; trainees who have lived experience etc. All participants needed to be able to complete the interview and survey in English, agree to be audio and/or video recorded, and able to attend two 2-h training sessions virtually through Zoom (i.e., needed to have the technology to participate). Individuals were eligible to take part regardless of their level of knowledge and familiarity with POR.

Participants were recruited in a variety of ways to strengthen the diversity of the research roles and positions of the stakeholders, applicability of the training workshops, and transferability of the results. We recruited locally within the co-authors’ institutions (e.g., through each internal research, trainee, and family engagement programs) and through collaboration with several Canadian childhood disability research centres, networks, and groups (e.g., through their newsletters and internal communication channels). We also utilized various social media strategies, such as through Facebook and Twitter, to recruit potential participants.

### Training workshops

Two groups were created based on availability and stakeholder identity to ensure representation from each of the previously mentioned stakeholder groups, an important feature of the training design, allowing for a mix of types of participants to contribute ideas and learn from one another during the guided discussion. Each group was offered training across two, 2-h workshops. Each workshop was delivered online via a secure Zoom link and included watching two of the recorded simulation videos and a facilitated discussion after each of the simulations. An overview of the training program is presented in Table 1, including the premise of the simulation and key learning points of each of the training videos.. Additional information about the recommended simulation process and further details about the choice of simulation and learning objectives can be found in the Additional file 2: Table S1 and online facilitator guide which is freely available online for ease of implementation into practice [32]. A minimum of 45 min per simulation is recommended in our facilitation guidebook, which provides sufficient time for discussion, reflection, idea generation, and planning, as well as a short break between each simulation. Both groups completed the workshops in the same order, viewing the same videos during workshops one and two. Participants were provided with an e-gift card at the end of the study.

**Table 1:** Overview of the training program

Title	Training Workshops			
	Training Workshop 1		Training Workshop 2	
	Finding a Family Partner	Partnering to Set Research Objectives	Reviewing Results	Navigating Concerns about KT
Premise of the Simulation	A researcher meets with an engagement specialist* to discuss how to find a family partner to include on a grant proposal that the researcher is submitting next week	A researcher has just received a large grant and is meeting with 3 family partners for the first time to obtain feedback on the project’s research objectives	A researcher meets with a physical therapy student who is also a former client to get their thoughts on the results of data analysis that is being conducted by the research team	A PhD student and their supervisor are meeting online with a family partner to discuss sharing their study’s results. Tensions arise when the family partner asks to share the results with her son’s principal, but the researchers want to wait until the study is published in an academic journal
Key Learning Points of the Simulation	Prompts learners to examine the value of building rapport and trust through time management, and the importance of setting the stage before seeking a family partner	Explores what happens when there is a discrepancy between the desired outcomes of family partners and researchers and highlights the importance of articulating the hopes and expectations of all team members at the outset of the project	Illustrates the multiple identities family partners bring to the research team, and if ignored or unwelcomed, the partnership may feel inauthentic. Emphasizes the importance of discussing roles and expectations of partner contributions and the value of lived experience	Explores ethical considerations of co-authorship and the need to discuss expectations at the beginning of a partnership

KT, knowledge translation

*The term *engagement specialist* is used to represent a staff member at an organization whose role is to support engagement opportunities between researchers and patient and family partners within the organization

Two trained facilitators (KP, BD) from the research team co-facilitated the training workshop and one study team member (SM) was available for technical/logistical support during the training. KP is director of academic affairs and simulation lead at Holland Bloorview Kids Rehabilitation Hospital and BD is a parent who was trained through the Holland Bloorview Family Leadership Program to co-facilitate simulations. BD was compensated for her time as a co-facilitator and a co-investigator on this project per institutional guidance. BD’s role was important to ensure the appropriate delivery and debrief of the training workshops to a diverse group of stakeholders that included parents and caregivers. Despite our efforts to include a youth partner facilitator, to our knowledge, no youth leaders who are trained in simulation facilitation exist at Holland Bloorview and it was beyond the scope of this pilot project to train youth leaders to facilitate the training workshops.

### Data collection methods and analysis

This research occurred in two phases. Phase 1 (quantitative strand) included a pre-training survey, the training workshops, and a post-training survey. Phase 2 (qualitative strand) included two qualitative focus groups. Qualitative reflections by workshop co-facilitators were also collected after each training session. Investigators on the team with qualitative, quantitative, and/or mixed methods experience (MP, SM, GK) were involved in this process. SM led the analytic process, guided by MP, and both worked closely with a youth leader (JK) who offered both lived experience and qualitative research experience during data analysis phase to better understand the significance and relevance of the responses.

*Demographic information.* Demographic information was collected from all participants in the pre-training survey. Questions included age, role, employment status, education, and institutional affiliation. Descriptive statistics were used to analyze numerical demographic data.

*Pre and post training surveys.* Investigator-developed pre/post training surveys were created using the online survey platform, REDCap [33]. Evaluation questions were developed to address the first two levels of The New World Kirkpatrick Model (NWKM), where level 1 (reaction) considers the immediate response of participants and “the degree to which participants find the training favorable, engaging and relevant to their jobs” [34, (para4)]. Level 2 (learning) in the NWKM addressed “the degree to which participants acquire the intended knowledge, skills, attitude, confidence and commitment based on their participation in the training” [34, (para5)]. The survey questions thus examined the extent that participants’ knowledge, attitudes, and self-perceived ability related to the challenges identified in the simulations changed from before to after the training on a scale of 1 (not at all) to seven (to a very great extent).

The pre-training survey was sent to participants via email approximately one week prior to the first of two training workshops. The post-training survey was sent to participants via email approximately one week after the second training workshop. It included the same set of questions addressing the extent of participants’ knowledge, attitudes, and self-perceived ability as the pre-training workshop survey, but also included questions about acceptability, appropriateness, and feasibility of the training workshops using the following three scales: Acceptability of Intervention Measure (AIM), Intervention Appropriateness Measure (IAM), and Feasibility of Intervention Measure (FIM) [35]. Each scale contains four questions related to the construct. All three scales show acceptable reliability (alpha = 0.85 for acceptability, alpha = 0.91 for appropriateness, and alpha = 0.89 for feasibility) [35]. Pre- and post-survey data were analysed using descriptive and inferential statistics. Wilcoxon Matched-Pairs Signed Rank Test was used to compare pre/post workshop scores in knowledge, attitudes, and self-perceived ability to engage in POR. The quantitative findings helped to inform the development of the focus group interview questions by indicating where additional depth was needed to better understand the quantitative findings. Given the sequential nature of this mixed methods design, we reflected on the results of both the quantitative and qualitative strands during the interpretation phase, and then integrated these data strands together at that point of analysis and interpretation.

*Focus groups.* All simulation training participants were invited to take part in an online focus group via Zoom which explored key ideas identified from the pre/post survey and were audio and video recorded. Focus group questions were developed from survey findings. Questions were framed based on the three constructs of primary interest: knowledge (e.g., Can you please describe anything you learned about authentic and meaningful collaboration in research during the training workshops?), self-perceived ability (e.g. Something that came up frequently in the open-ended questions was the importance of mutual respect and communication. How can we build mutual respect?) and attitudes (e.g., How likely are you to use what you learned in the training workshops in your upcoming projects, work or roles related to patient-oriented research or patient engagement in research?). Follow up questions about intent to apply the learnings from the training workshop and barriers and facilitators were asked. The focus group recording was transcribed and analysed using the constant comparison method outlined by Onwuegbuzie et al. (2009) where the transcribed text is first open-coded (i.e., small units of data grouped together) and a code or descriptor is added to each small grouped unit of data (e.g., “Need for Training") [36]. JK, SM, and MP met frequently during this stage of the coding. The assigned codes were then categorized (i.e., axial coding) [32] by SM with the support of JK and MP. For example, “Need for Training” was combined with other contextual factors that influenced the implementation of POR, e.g., top-down pressures that influence the time and training available for people who engage in POR. The full team was then engaged in a final discussion of the categories and further category development through review of the written findings.

*Co-facilitator reflections.* Co-facilitators of the training workshops provided typed reflections about the nuances and interpretations of the process of co-facilitating the training workshop. Specifically, we were interested to know if procedural components (e.g., timing, delivery pace) of the training workshops need to be adjusted in future sessions to improve the overall usability of the training workshops for future use. Documents were reviewed by SM to understand potential barriers and recommendations that could inform future training sessions. No coding was done for these reflections; rather the focus was on a descriptive summary, inclusive of all reflections, to support a comprehensive evaluation of the training workshops.

## Findings

The findings of this research are presented in four sections below. We first describe characteristics of the participants that took part in this study. We share details from the focus group discussions to provide the context regarding POR within the participants’ work. Next, we present participants’ training experiences gathered from pre/post training surveys and focus group discussions. This included perspectives on the content of the simulations, the structure/formatting of the training, and the emotional reactions that participants experienced. After this, we share insights from both the pre/post training surveys and focus group discussions on the training outcomes regarding participants’ knowledge, attitudes, abilities, and intent. Finally, we provide recommendations for revising the simulation content and presentation, additional training, and creating systems-level change, as well as insights from the training facilitators reflecting on this experience.

### Participants

Sixteen individuals took part in this research study, the majority of whom were female (n = 14, 87.5%), had completed a graduate degree (n = 13, 81.3%), and were geographically located in Canada (n = 14, 87.5%). Most participants (n = 10, 62.5%) had not received any formal training about POR (e.g., educational workshops, webinars, courses, modules, etc.) that focused on engaging youth with disabilities and/or parents of children with disabilities or focused on engaging with research teams. Of those who had received formal training (n = 6, 37.5%), the most frequently reported training was the Kids Brain Health Network (KBHN)/CanChild McMaster Family Engagement in Research Course. Only five of sixteen participants had previous experience using simulation as a training tool, however, these experiences were not specific to POR. See Table 2.

**Table 2:** Socio-demographic information of participants who took part in the training

Variable	N (%)
*Participant sex*	
Female	14 (87.5%)
Did not respond	2 (12.5%)
*Participant age range*	
20–29	4 (25.0%)
30–39	4 (25.0%)
40–49	5 (32.2%)
50–59	3 (18.8%)
*Highest level of education completed*	
Some high school	1 (6.2%)
Graduated college/university/technical school	2 (12.5%)
Graduate degree	13 (81.3%)
Current role/position*	
Researcher	5 (32.2%)
Student/trainee	7 (43.8%)
Parent/caregiver	5 (32.2%)
Clinician	3 (18.8%)
*Current employment status*	
Unemployed/unpaid work/volunteer/student	4 (25.0%)
Part time	3 (18.8%)
Full time	6 (37.5%)
Multiple roles/positions	3 (18.8%)
*Geographic location*	
Canada	14 (87.5%)
Outside of Canada	2 (12.5%)
*Institutional Affiliation*	
Academic teaching hospital	3 (18.8%)
University	9 (56.2%)
Research center	1 (6.2%)
Does not apply	3 (18.8%)

*Some participants have multiple roles

In their previous research studies, many participants had experience(s) with research projects that engaged youth/family partners in one or more of the following study phases: study conception / study design (n = 6) (e.g., helping to create study team or advisory committee); during the study (n = 10) (e.g., developing research questions, providing feedback on study tools and recruitment methods, and collecting data); in completing the study (n = 2) (e.g., having a role in data analysis, writing results or other documents); and after the study (n = 9) (e.g., sharing findings via presentations and helping to implement findings). Only two participants reported that the youth/family partner was involved in systematic or other types of reviews.

In their qualitative interviews, participants situated their experiences within the POR context, describing barriers to and facilitators of authentic and meaningful engagement and concluded with the need for additional training. In this section, we summarize the POR context, describe participants’ experiences of the training, illustrate outcomes, and impact of the training, and provide recommendations for use of the simulations and next steps.

### Participants’ contexts in which POR occurred

In both the open-ended survey responses and the focus group discussions, participants described the ‘bigger picture’ context in which engagement occurred. From the open-ended survey responses, participants situated themselves in a culture that values POR as a way of promoting the inclusion of patient partners throughout the research process. Participants named the lens of "nothing about us without us" to ensure that research includes patients and families to address questions that are relevant to them. In the focus groups, authentic and meaningful engagement was described as being guided by principles such as diversity and inclusion, support, mutual respect, honesty, openness to share, accessibility, and having expectations clearly defined and/or negotiated. Authentic engagement according to one focus group participant meant “*being authentic is yeah being kind, it’s being thoughtful, it’s saying, gee that’s a great question I have no idea. Like let me think about that, let me get back to you, I don’t know the answers*” (Participant 104, Research Staff).

Participants described multiple challenges to meaningful engagement, including the time and effort it takes. Noted barriers included a lack of researcher time and resources to foster authentic connections, top-down pressures that can limit the diversity of caregivers/family partners being asked to engage, lack of funding, and lack of skills and training, which impacts the research as well as the individuals involved: *“you’re rushing too much to kind of know how that affects someone”* (Participant 102, Research Trainee). A variety of skills were described as important to conduct authentic and meaningful research engagement (e.g., clear and transparent communication; active listening; honesty; mutual respect; empathy; an open mindset; and an interest in, appreciation for and valuing all experiences and ways of knowing) which are not typically taught in research training programs.

These challenges and barriers shed light on the need for ongoing training, which should start early in researcher’s schooling and career (e.g., in graduate degree programs) and include training on communication, self-awareness, and power differentials: *“It’s okay that people are good and bad at things. You don’t have to be good at everything. But understanding that…if I’m not a good facilitator then maybe I shouldn’t run it [the meeting]”* (Participant 105, Family Partner). Training via simulation was described as an important way to potentially bridge these gaps: “*I honestly think that really having every parent-patient go through and see these processes and hearing from the other sides of things would be more beneficial for each party*” (Participant 101, Family Partner).

### Participant training experience

Overall, the training was well received by participants and their experiences were positive, for example: *“as a newer PhD student, I feel like I could take this patient engagement course ten times over and learn like a little bit more along the way”* (Participant 106, Research Trainee). Participant responses were synthesized from multiple sources, including the pre/post training survey and the focus group discussion. In the post-training survey, participants rated their perceptions of the simulation training on measures appropriateness, acceptability, and feasibility where higher scores indicate greater appropriateness, acceptability, and feasibility, respectively. [35] Participants scored the simulation training high on all three measures, as indicated in Table 3 below. While there is currently no validated cut-off point for these measures, feasibility was lowest rated by participants, indicating there may be practical challenges or barriers to implementing the training into practice.

**Table 3:** Acceptability, appropriateness, and feasibility of the training

Measure	Mean (Standard Deviation) (n = 15)
Acceptability of Intervention Measure (AIM)	4.7 (0.5)
Intervention Appropriateness Measure (IAM)	4.5 (0.7)
Feasibility of Intervention Measure (FIM)	4.4 (0.6)

For AIM, IAM, FIM measures, see Weiner et al., 2017. Total score for each measure = 5

In the focus groups, participants elaborated on the training experience, expanding on the content of the simulations, the structure/formatting of the training, and the emotional reactions they experienced. These ideas will be further described in the following section.

*Content of the Simulation Videos.* Participants felt that the content presented via the training was applicable to a wide variety of participants and projects: *“I think the videos were good in maybe reaching a large breadth of people who may be in the beginning stages of patient engagement to the far end of it”* (Participant 106, Research Trainee). Given that the simulation content was specific and focused on challenges that can arise when engaging in research, participants felt that this was a helpful lens to learn from: *“the fact that the training scenarios were kind of like purposefully bad or wrong makes it easier to highlight the things that are wrong…if the conversation had been perfect or near perfect, picking out the elements of what make a good interaction, it’s harder when it’s kind of gone well”* (Participant 103, Researcher). While it was helpful to have exaggerated negative situations depicted in the simulations, they were often described as ‘polarizing’. Participants agreed that the simulation titled ‘Navigating Concerns about KT’ was the most impactful because it provided a realistic scenario in which the problem was not immediately apparent: *“It provided a scenario that some people were like…I don’t see what’s wrong with this…it allowed us the opportunity to show okay but there’s so much more going on behind the scenes for researchers and research partners that maybe we don’t know”* (Participant 201, Family Partner).

The content of the simulation videos also generated a variety of different emotions, such as disbelief, discomfort, and embarrassment, which could then be explored during the group discussion and debrief: *“It’s like watching something happen in slow motion. You’re like ‘no that’s the worst idea you could possibly think of, stop’. You feel terrible for everybody in the situation.”* (Participant 105, Family Partner). Specifically, the final simulation (‘Navigating Concerns about KT’), which was described as more nuanced, was quite emotionally impactful and participants explained how it felt more realistic: *“Yeah, it was a bit more nuanced to allow I think some of that really messy stuff that does happen”* (Participant 104, Research Staff). Watching the videos allowed participants to put themselves into the scenario, understand the depth of the scenario and how it might apply to their own work: *“it just it really touched close to home with where I’m at in my stage in my career as a graduate trainee and recognizing that those situations are probably something that can definitely happen”* (Participant 204, Research Trainee). Moreover, because the training participants are not in any way connected to the people involved or invested in the scenario itself, they could step back and view the scenario more objectively and better understand its impact. Two participants called this a ‘third eye’ which meant being able to look at the scenario from a distance without themselves in it. Having these types of emotional interpersonal challenges arising in research engagement are not always noticeable in the moment: *“by seeing the really uncomfortable videos it can show people when you’re rushing through something, what that emotionally like the affect is”* (Participant 102, Research Trainee).

*Structure of the Training.* Participants described that a significant strength of the training experience was how the training was structured. The use of small group discussions and the ability of the facilitator to reframe the group discussion had a positive impact on their learning: *“we all felt we had a chance to talk and say what our opinions were and it’s a little bit easier to share I think when you’re in a smaller group”* (Participant 203, Family Partner). Additionally, the group discussion format provided space to listen to multiple perspectives from different roles (e.g., parent, researcher, trainee) and this was impactful for both family partners: *“I found it extremely valuable to hear other people’s opinions of how they were viewing the same scene as I was. How all of our life experiences make us see or experience that moment in a different way”* (Participant 201, Family Partner) and researchers: *“just hearing someone else either talk about the same point that I had but from a different perspective. It absolutely contributed to my learning”* (Participant 103, Researcher).

The structure of the training was particularly important because of the facilitated group discussion and participants suggested that the training would not be amenable to a self-paced, individual, online module format (i.e., not synchronous and without a facilitator): *“I worry about how other people might interpret that or how they might go about with addressing it without kind of knowing about the foundations of let’s say family engagement”* (Participant 204, Research Trainee). The simulations paired with facilitated discussion was important to creating a strong learning environment where participants could feel heard and learn together: *“I think that the interplay between observing, reflecting, conversing was really the most powerful thing about it. Was being able to hear other people’s perspectives and to hear your own voice in those perspectives but also to hear different voices.”* (Participant 103, Researcher).

### Training outcomes regarding knowledge, attitudes, ability, and intent to use the training

Training outcomes regarding participants’ knowledge, attitudes, abilities, and intent were gleaned from both the pre/post training surveys and followed up in the focus group discussions. Participants scored measures related to knowledge and ability significantly higher on the post-training survey, indicating an improvement in knowledge (*W* = 4.00, *p* < 0.001) about how to promote meaningful and authentic engagement, as well as confidence (ability) (*W* = 5.00, *p* = 0.012) to engage with different groups on research teams. There were no additional significant differences in other outcomes related to attitudes or ability (see Table 4). There was also no significant increase in participants’ attitudes towards the importance of building authentic and meaningful research partnerships or the use of evidence-based strategies related to engagement in research.

**Table 4:** Participants’ self-perceived knowledge, ability, and attitudes pre- and post- training

Outcome Construct	Median (Pre)	Median (Post)	*W*-Statistic	*p *value
**Knowledge A:** I know how to conduct patient engagement in research in a meaningful and authentic way	4	6	4.00	0.001*
**Knowledge B:** I understand the value in partnering with researchers or clients/family on research teams	5	7	5.00	0.024*
**Ability A:** Based on my current ability, I am confident that I can collaborate with others (researchers or clients and families) on research teams	5	6	5.00	0.012*
**Ability B:** Based on my current ability, I am confident that I can communicate with others (researchers or clients and families) on a research team	6	6	5.00	0.075
**Attitudes A:** Using evidence-based strategies related to patient engagement in research is important to me	6	7	9.00	0.052
**Attitudes B:** Building authentic and meaningful partnerships in research is important to me	7	7	3.50	0.129
**Intent A:** In the future, I intend to engage with researchers or clients and families on research teams in the future	6	7	9.00	0.824
**Intent B:** In the future, I intend to use patient engagement knowledge and skills to meaningfully and authentically engage with researchers or clients and families on research teams	7	7	8.00	0.299

Responses regarding knowledge, ability, attitudes, and intent were measured on a 7-pt Likert scale

*p < 0.05

To help explain these findings, we asked focus group participants to expand on their experiences and the impact the training had on their learning. Discussions indicated increased awareness and knowledge around specific topics and issues, such as: the need for there to be more than one family member on a research team; the idea that there will be moments of ‘healthy tension’ on research teams and that engagement takes a lot of time to do; and the need to prioritize building long-term connections. For example, in reference to the simulation titled ‘Navigating Concerns about KT’, one participant explained how the content helped them to understand the importance of communication between researchers and family partners working together on research teams: *“It just reinforced how much communication is so important and that everyone who comes to the team—I don’t think there are people who are doing bad family engagement *per se* like they don’t want to but sometimes it really comes across that way”* (Participant 204, Research Trainee). While another participant explained: *“These sims showed me that there’s an additional step that needs to be taken that we both need to be more open with each other of what is happening outside of this research project”* (Participant 201, Family Partner).

Participants also described the impact the simulation training had on the awareness of their own behaviours, indicating that the training acted as a self-check tool, or something they could use to validate their own behaviours. For example, one family partner stated: *“I find it almost like a sensitivity course where it’s just you have to be aware”* (Participant 101, Family Partner). Another participant explained how the training helped them understand how they might respond in a similar situation: *“I think they just exposed you to something that maybe you haven’t seen before and how maybe you would respond in that situation”* (Participant 107, Research Staff).

Survey results showed no significant difference pre- and post- training in participants’ intent to engage with researchers and families in the future nor on their intent to use their knowledge and skills gained to engage with researchers or clients and families meaningfully and authentically on research teams in their future work (see Table 4). The open-ended survey responses and focus group discussions, however, provided a more nuanced insight about participants’ intent around the simulation content and the training experience. Multiple participants indicated how they might incorporate their learnings into their current or future work (e.g., in the planning and conduct of research), including sharing knowledge with others (e.g., other parents, supervisor), advocating, and supporting their family. Others indicated the skills they gained from the simulations, such as taking time to plan for engagement, setting up expectations, and communicating openly and clearly. The simulations were helpful to understand what the process should look like and what happens when things do not go as planned. Participants also raised the multiplicity of roles (i.e., some are family members and researchers, while others have clinical backgrounds and research roles) and the potentiality for knowledge and skills gained through the training could inform their interactions beyond pediatric disability research. Similar comments were raised in the focus groups related to how they would like to take the knowledge gained (e.g., related to the content of the simulation videos and discussions) and integrate it into their current engagement practices: “*my intent is to do exactly what we were just talking about a few moments ago. About, you know, building engagement with the research program as opposed to specific research projects. Cause I think there’s value in creating those long-lasting relationships”* (Participant 103, Researcher); and with the training videos themselves: *“I think in the future down the line I would love to do it within as a team exercise with the researchers and family partners”* (Participant 204, Research Trainee).

### Participants’ recommendations

*Simulation Content and Presentation.* Participants suggested that the video content be more subtle or nuanced regarding what was ‘wrong’ to elicit deeper conversations around more complex situations: “*I thought maybe making a video that’s a little more subtle could spike those conversations potentially*” (Participant 108, Research Trainee). Additionally, participants suggested different topics for the simulations to cover, including navigating longitudinal relationships between researchers and family partners. The simulation videos were filmed prior to the COVID-19 pandemic and a disclaimer regarding this context would situate the videos in a way to not distract from the content (e.g., the actors were sitting close together, not wearing masks etc.): *“you probably do need a bit of a disclaimer in the short term that you know these were filmed pre-pandemic—it was jarring…it definitely contributed to my uncomfortableness in the first couple session”* (Participant 103, Researcher). The disclaimer could also indicate that the videos were purposefully filmed as challenges to elicit an emotional response and deepen discussions for learning. Finally, participants suggested having a physical resource or summary of the key take-aways provided at the end of training would be helpful for long-term recall.

*Expanded Training.* Participants indicated that there is a need for training beyond just researchers and family members, but also for individuals in higher-ranked positions within academic settings: “*Because ultimately those are gonna be people that are there then supervising who’ve done it, if that makes sense*” (Participant 103, Researcher). Hearing perspectives from all sides is important, and this includes those who are in positions of decision-making power and can influence change in the research/academic setting: “*I think everybody who wants to participate in any sort of like research should actually have to go through this*" (Participant 101, Family Partner).

*Systems-Level Change.* Participants explained that time limits the depth or degree to which authentic and meaningful engagement can be fostered in a practical sense. For this to change, one participant explained that it is going to take more than just researcher and family partner training: *“we really need a big institutional change, which I don’t know how that happens in order to provide the time that’s necessary for those relationships to be built”* (Participant 203, Family Partner).

### Facilitator reflections

After completing the simulation training, the training facilitators were asked to reflect on their experience leading the workshops. Both facilitators reflected on participants’ high level of engagement in the training, investment in the topic, as well as the richness of the group discussions. While participants could take part in the group discussion orally and/or through the chat function in Zoom, we noticed that not all participants contributed equally, and more family participation would have been optimal. Facilitators commented on the challenges with online simulation training compared to their previous experiences with delivering in-person simulation training. While online training affords more convenience and ease of access for most participants, there were challenges with its delivery. For example, virtual participation made it harder for the facilitators to ‘read the room’ and pick up on non-verbal communication techniques. Facilitators indicated that some cues were not possible to pick up on and some participants had their cameras off, so it was challenging to connect at times.

## Discussion

The development of tools and resources to build capacity in patient engagement are core principles of Canada’s Strategy for Patient Oriented Research (SPOR) [1]. In this article, we expanded upon our team’s previous work by describing the delivery and evaluation of the four simulation training videos to build capacity in POR [8]. To our knowledge, no previous POR training programs exist that use simulation as a training tool. Of the training programs currently available related to POR, few, if any, have published evaluations of their programs or courses.

Participants described a variety of skills that are important to engage in POR, but which are not typically taught in research training programs. These skills included clear and transparent communication; active listening; mutual respect; and empathy, among others. For example, listening and effective interpersonal communication, which is largely overlooked [37], can be enhanced through the use of simulations [38]. This is aligned with the general simulation literature where simulation is supported as an effective tool to enhance communication skills, which can then be transferred out of the simulation space into practice [39, 40]. Skills such as communication, teamwork and group processing, and creating safe and respectful environment were found by Frisch et al. (2020) to be key patient-oriented research competencies for health researchers, yet the barriers to engagement described by participants in this study shed light on the need for ongoing training in these areas [41].

Simulation is a unique tool that can be used to effectively teach and support the development of some of these ‘soft skills’ that may impede authentic and meaningful engagement. Participants explained that there were multiple barriers to authentic and meaningful engagement (e.g., time and extra effort/thought needed; lack of organizational policies and funding to compensate patient partners; and limited project timelines such as for graduate student theses). These barriers underscore the importance of transparent communication and clear expectations, a finding that is consistent with our previous work [8]. Future research in this area may wish to explore simulation as an educational tool to improve other ‘soft skills’ beneficial to engage in research partnerships, such as confidence, which simulation has shown to improve [42].

Through our evaluation of this training, we found the four simulations to be acceptable, appropriate, and feasible by participants, and overall, participants’ reactions to the training were positive and they provided constructive feedback for future use. Our findings align with the general simulation literature which shows that learners generally prefer simulation as an educational tool compared to other forms of educational delivery, such as traditional classroom lectures or online learning [42]. In the current study, we found significant differences between pre and post survey responses on participants’ self-reported knowledge and ability but not their attitudes related to POR. As most participants in our study indicated a high level of previous experience engaging in POR and some even had previous POR training, the fact that we did not find a difference in attitudes related to POR is not surprising. It is likely that participants who took part in the current research study were already highly engaged in POR and had an interest in building their capacity to engage in research within collaborative, interdisciplinary groups. There were many components of our simulation training that participants liked, including the content, which was focused on the challenges and provided a helpful lens to learn from. Participants in the current study agreed that the simulations provided a space to listen to multiple perspectives, and this is consistent with findings from simulation studies in the broader literature [26, 43] as essential learning typically occurs in the debriefing stage [44].

While participants liked that the simulation topics and content were exaggerated to highlight challenges that may be experienced, participants also felt that the simulation where the problem was not immediately apparent was the most impactful. Using Mezirow’s Transformative Learning Theory (TLT), Briese et al. applied the 10 phases of TLT to simulation, where the first phase is exposure to a ‘disorienting dilemma’ in the pre-briefing and scenario aspects of the simulation [45]. The dilemma is intended to get learners to think about a problem that needs to be solved and understand the resources that might be needed to solve it. This raises an interesting opportunity for future research to explore how the complexity and intensity of the scenario’s ‘dilemma’ impacts learning.

The exaggerated simulations elicited a lot of emotions yet also provided participants with a way to disengage from the content and scenario so that they could ‘step back’ and view the simulation more objectively. Given the realistic nature of the simulated scenarios, learners can be engaged emotionally [46]. This is somewhat in contrast to our findings in which the learners felt the simulation offered a somewhat objective position given that the situation was not ‘real’. Research linked to this concept indicates that the value of simulation lies in the fact that actual risk is minimized—there is no real risk because the scenario is not real [46, 47]. Despite providing a strong emotional reaction, learners were able to distance themselves from the situation, meaning that any worry about the outcome of the scenario was removed. This tension between subjective emotion generation and objectivity of learning experience should be explored in future research.

### Strengths & limitations

The study team included multidisciplinary researchers and clinician-researchers, and three members were patient/family partners who were included at all phases of the research process, including data collection (i.e., patient/family partner simulation training co-facilitator) and data analysis (e.g., reading transcripts; interpretation). Unfortunately, we were unable to recruit youth and young adults for the study and therefore it is difficult to know how they would perceive the training, despite youth being involved in the simulation development. In the future, we would like to explore the use of the training specifically with youth by partnering with a youth-based POR training program at our local children’s rehabilitation hospital. As we cannot determine the effectiveness of the training for youth, we would like to introduce and evaluate the training as part of the hospital’s current onboarding training within its youth volunteer program.

This study used pre-recorded, fixed simulations which are limited in their methodology. With live simulations, the facilitator is afforded the opportunity to take the learning where the participants want it to go and allows for more subtle or deep conversations. With video-based simulation, the learning primarily happens during the debrief discussion. Some studies suggest that live simulations may result in better learning when compared to video-based simulations although this is not conclusive [48]. Because most participants were already experienced with POR, there is a risk of selection bias as participants were likely committed to the topic and very interested in the skill building component of the study and thus more likely to participate in the study [49]. In the future, we would like to explore the simulations with individuals who have no previous experience of POR. Moreover, further bias could have been introduced through the provision of an honorarium. However, we believe it was important to compensate people, especially those with lived experience, for their time and knowledge.

### Reflections on patient engagement in this research study

Similar to our previous work, youth and family partners on the study team were engaged throughout the research process in ways that were reflective of their interest, skills, and strengths [8].Partners were provided choice with how they wanted to engage and what aspects of the research they wanted to engage in through the use of a team charter. While this work was conducted completely online, providing an accessible way to meet as a team, we experienced challenges during the data analysis phase with sharing study documents that could not be sent via email with team members outside of the primary author’s organization. This made it difficult to collaborate with youth and family partners who are not otherwise affiliated with the organization during the data analysis process. Organizational challenges such as data storage and access present significant barriers to equitable engagement and inclusion. To overcome this, we found it especially important to be creative, resourceful, and tenacious, and to collaboratively develop ethical and accessible solutions. Creative solutions were found through team members thinking ‘outside of the box’, for example, to overcome challenges with organizational cloud-based access, our youth partner, JK, found that their graduate school email worked better than their work affiliation. While we could have foregone JK’s involvement in data analysis given the challenges in accessing the data, it was important to the team that lived experience perspectives were embedded throughout the project. Additional strategies included consulting engagement specialists on our team to determine the best strategies for recruitment and the best day/times to hold online events.

### Future directions

Simulation is a powerful tool to build capacity in meaningful and authentic POR, and as a team, we plan to continue to explore how simulation can be integrated into different learning contexts. The simulation training presented in this paper was found to support participants’ knowledge and ability to engage in meaningful and authentic engaged research and we encourage other teams to integrate this work into current or new training models. For researchers, family members, and organizations interested in using the simulations for capacity building around authentic and meaningful engagement in research, based on our experiences, we recommend: (1) facilitators have experience in running small group discussions and/or experience with debriefing conversations, which is in line with the International Nursing Association for Clinical Simulation and Learning Standards of Practice [44, 50]; (2) a co-facilitator model (i.e., lived experience and researcher or educator) for delivery of the simulations, as well as a person available for technical/logistical support during the training; (3) ‘pre-brief' with the co-facilitators to test materials, video sharing, and decide on roles during the training as well as a clear understanding of the learning objectives for the simulation as different objectives could be addressed in any one simulation; and (4) having a constellation of perspectives among participants (e.g., researcher and family) is important, adding to the value of the debrief and potential to foster empathy. Research on family engagement in research that uses simulations should focus on the mode of delivery of the simulations (i.e., comparing digital and live simulations), determine whether subtle or more exaggerated content affects participants’ learning, and exploring which other topics about family engagement in research should be covered in future simulation development.

## Conclusion

We found the suite of simulations developed by our research team in 2019 to be a positive and impactful way for collaborative research teams to develop knowledge and ability to engage in authentic and meaningful POR. Despite its inherent limitations, online simulation training that uses a co-facilitator model where researchers and family partners learn together in a synchronous environment resulted in positive feedback. We believe this is because of the opportunities to learn from people who have other roles and experiences and engage in facilitate discussion about common and emotionally powerful scenarios in a safe environment. Recommendations for future work include covering different content with varying levels of nuance and offering the training to individuals beyond the research team, including those higher-ranked academic positions.

## Supplementary Information

**Additional file 1**. **Table 1:** Overview of the simulation training program process.

**Additional file 2**. **Table 2:** GRIPP2 long form.

## Data Availability

Not applicable.

## Abbreviations

AIM: Acceptability of Intervention Measure
IAM: Intervention Appropriateness Measure
FIM: Feasibility of Intervention Measure
POR: Patient-Oriented Research
SP: Standardized Patients
