# Supplementary material for: Delivery and evaluation of simulations to promote authentic and meaningful engagement in childhood disability research
Source: Res Involv Engagem. 2023 Jul 18;9:54. doi: 10.1186/s40900-023-00468-9 (PMC10353094; doi:10.1186/s40900-023-00468-9)
Supplement: Supplementary file 2 — Additional file 2. Table 2: GRIPP2 long form. [file 40900_2023_468_MOESM2_ESM.docx]

Supplementary Table

*Overview of the Simulation Training Program Process*

|  | **Training Workshop Agenda** |
| --- | --- |
| **Welcome & Opening Remarks** | - Provide instructions on the functions of the communication platform (e.g., use of video, mute, and chat functions). - Introduce purpose of gathering. - Everyone introduces themselves. |
| **Overview the Simulation and Debrief Process** | - Provide an overview of what will happen during and after the simulation, including the debrief process. - Provide an opportunity to ask questions. |
| **Watch Video 1**  **& Debrief** | - As a group, read the learning objectives and scenario presented on screen. - Watch the first video of the training workshop.   - The facilitator will display the video on screen and instruct learners to watch and not take notes.   - The facilitator will run the same video a second time. This time, they will instruct the learners to note anything that happens or anything that anyone says in the simulation that learner has an emotional reaction to. - Facilitators moderate a discussion with the learners to ‘de-brief’ about what was presented in the video.   - Facilitators will provide time for learners to note their thoughts and reflections and/or expand on their reactions.   - Simulation debrief will follow the plus-delta model* which centres around the following two questions:     - “What went well in the simulation?”     - “What could be improved?” - In applying this debrief model* specific questions were asked for each of the simulation videos and are included in the facilitation guides. For example, Simulation Two questions included:   - How do you think Dr. Jones and the three family partners felt throughout the encounter?   - What characterizes the communication between Dr. Jones and the family leaders?   - **I**f you were leading this conversation with family partners, what might you do differently? What difference do you think that would make? |
| Break | |
| **Watch Video 2**  **& Debrief** | - Watch the second video of the training workshop and debrief according to instructions for Video 1 above. |
| **Questions &**  **Adjournment** | - Provide an opportunity for learners to ask questions and share any final reflections. - Provide a reminder about next steps. |

* The plus-delta model was used to guide self-assessment of learning during the simulation training. See: Cheng, A., Eppich, W., Epps, C. *et al.* Embracing informed learner self-assessment during debriefing: the art of plus-delta. *Adv Simul* **6**, 22 (2021). <https://doi.org/10.1186/s41077-021-00173-1>
